# Aesthetic-Cosmetic Concern and Its Importance: A Questionnaire-Based Comparative Study of Posterior-Midline and Postero-Lateral Scars in Lower Back Surgery

**DOI:** 10.7759/cureus.103442

**Published:** 2026-02-11

**Authors:** Ajay Krishnan, Abhijith Anil, Shivanand C Mayi, Ravi Ranjan Rai, Mirant B Dave, Mikeson Panthackel, Arjit Vashishtha, Amritesh Singh, Preety Krishnan, Bharat R Dave

**Affiliations:** 1 Spine, Bhavnagar Institute of Medical Science, Bhavnagar, IND; 2 Spine Surgery, Stavya Spine Hospital and Research Institute, Ahmedabad, IND; 3 Orthopaedics/Spine Surgery, Pushpagiri Institute of Medical Sciences, Thiruvalla, IND; 4 Orthopaedics, Geetanjali Medical College and Hospital, Udaipur, IND; 5 Orthopaedics, University College of Medical Sciences and GTB Hospital, Delhi, IND; 6 Radiology, Stavya Spine Hospital and Research Institute, Ahmedabad, IND

**Keywords:** aesthetics, lumbar, minimally invasive spine surgery, scar, spine, surgery

## Abstract

Introduction

Literature concerning the aesthetic appeal of incisions following minimally invasive spine surgery is sparse. We aimed to address this gap in the literature and to understand the cosmetic preferences of patients with respect to the postoperative scars after spine surgery.

Objectives

This cross-sectional study aimed to identify patient preferences and aesthetic concerns related to surgical scars in MIST (minimally invasive transforaminal lumbar interbody fusion (TLIF)) and SOST (standard open surgical TLIF).

Methodology

A cross-sectional questionnaire-based study was conducted among people visiting an outpatient spine clinic to compare the aesthetic preference of patients. The study included 399 participants, with an age of 33.58 (12.82) years, consisting of 229 females and 170 males. The responses to the questionnaire with illustrative scars were analyzed with descriptive statistics to arrive at a conclusion.

Results

Among the participants, 238 expressed aesthetic concerns about scar appearance. A total of 153 (38.3%) respondents were more concerned about a scar on the back when compared to scars elsewhere on the body. A total of 306 (76.69%) individuals preferred the scar of surgical midline posterior TLIF (SOST) while 93 selected the MIST scar (p < 0.001). Notably, 139 of these participants (45.42%) changed their preference to MIST scars (p < 0.001) upon learning about the advantages of the modern MIST technique.

Conclusions

Patient-oriented aesthetic outcomes in surgery are essential. However, a lumbar spine surgical scar is of less concern than scars elsewhere on the body. Midline scars were noted to be more aesthetically pleasing than paramedian scars. Counselling regarding the advantages of modern minimally invasive techniques, however, can improve patient acceptance of the procedure despite the scar. Addressing patient concerns about scar appearance, psychological impact, and social implications should be integral to surgical decision-making and postoperative care.

## Introduction

Recent years have seen significant advancements in spinal surgery, with multiple techniques available to efficiently address lumbar spine disorders with fusion [1-4]. Lumbar interbody fusion (LIF) is a common surgical strategy that can be used to tackle unstable degenerative conditions, trauma, infection, and neoplasia. It can be performed by a dorsal approach (transforaminal lumbar interbody fusion (TLIF) and posterior lumbar interbody fusion (PLIF)) or by a ventral approach like anterior (ALIF), trans-psoas lateral (LLIF), or oblique lumbar interbody fusion (OLIF) [5-7]. Of these techniques, the most commonly used are standard open surgical midline posterior TLIF (SOST) and minimally invasive surgery TLIF (MIST). These two approaches are the workhorse surgical procedures in treating different lumbar spinal disorders [8-10]. While the SOST was the gold standard for a long time, MIST offers an alternative with decreased soft tissue disruption, blood loss, and faster recovery with equivalent long-term outcomes [9,11]. The postoperative scar is one aspect of lumbar spine surgery that is frequently overlooked and ignored.

In the context of surgery, the implications of a scar’s size and appearance for patients are enormous [12]. It is not just an issue of wound healing but can seriously affect a person’s confidence and mental health. For example, in surgeries like scoliosis surgery, where the scar is particularly long, it becomes part of your identity, even leading to the point of post-traumatic stress disorder (PTSD) [12-15]. The aesthetic outcome of these lumbar surgical incisions can also be crucial to how the patient perceives the outcome of surgery. Only a few studies have reviewed the impact of scars on patients in the current literature [16,17]. Multiple anecdotal reports claim that the MIST scar is often described as “better looking” or “less visible” than its SOS counterpart [18]. Although many articles might discuss the topic, most hospital websites flaunt it, and companies and conferences highlight it; more objective scoring or controlled studies must be conducted comparing these scars in order to make a definitive statement. To fill this void, a prospective study was conducted to objectively investigate people’s choices in the context of postoperative scar aesthetics. We aimed to determine respondents’ elective surgical approach in lumbar spine surgery based on anticipated postoperative aesthetics.

## Materials and methods

This survey was conducted from the 1st of September 2023 to the 20th of September 2023. Ethical approval for the study protocol was obtained per Maruti Healthcare Independent Ethics Committee (MHC IEC) guidelines. The study has been registered in the Clinical Trial Registry of India (CTRI) with registration number CTRI/2023/07/054902. Since no personally identifiable information was recorded and confidentiality was stringently protected, as per IEC, informed consent was not required to protect participant anonymity. Participants were recruited from the general population by convenience sampling if they were aged 18 or older and mentally sound, with no specific exclusion criteria for background or medical history other than previous spine surgery.

Data collection

**Figure 1:**
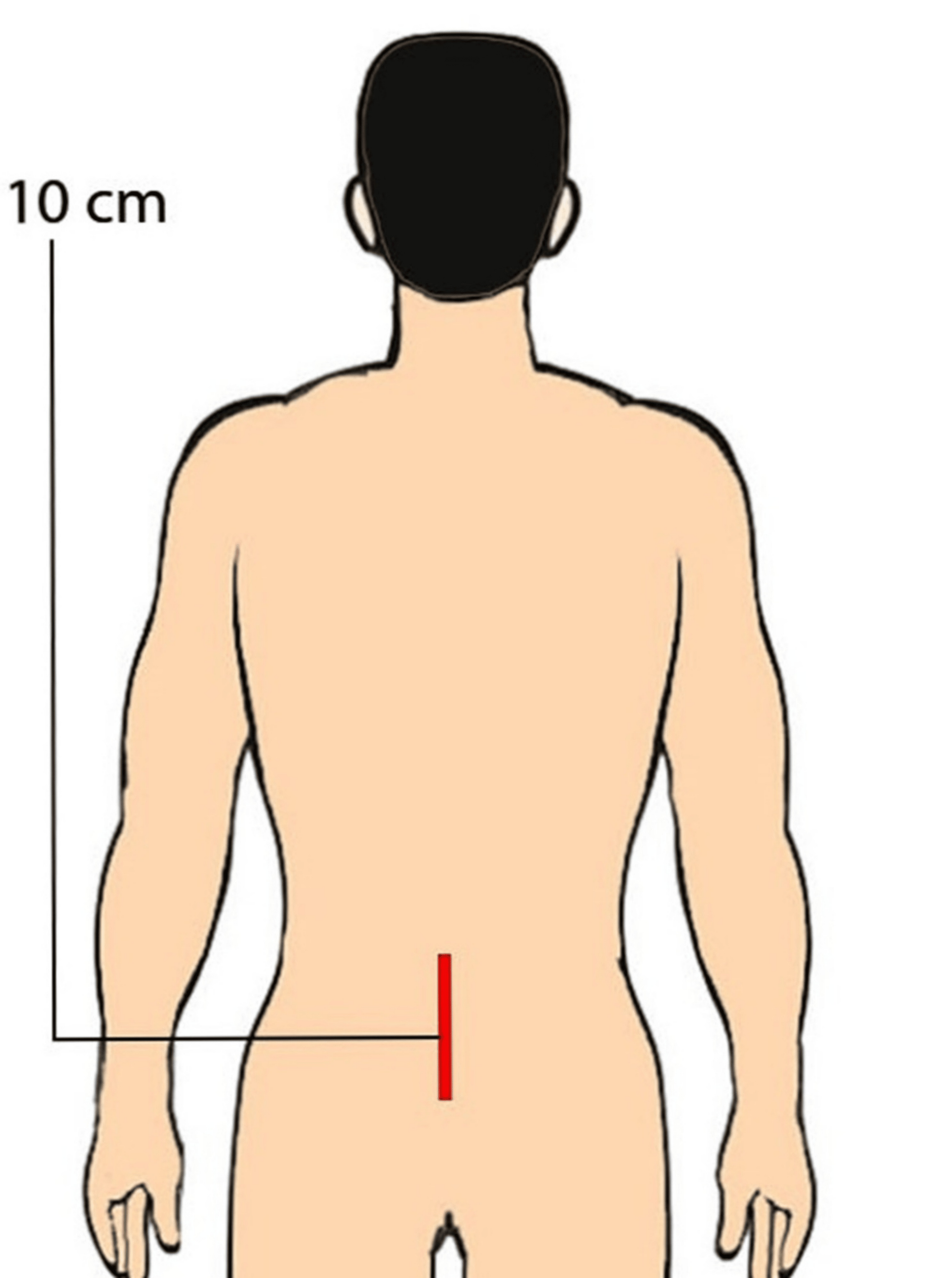
An animated picture showing a single incision scar on the lower lumbar level

**Figure 2:**
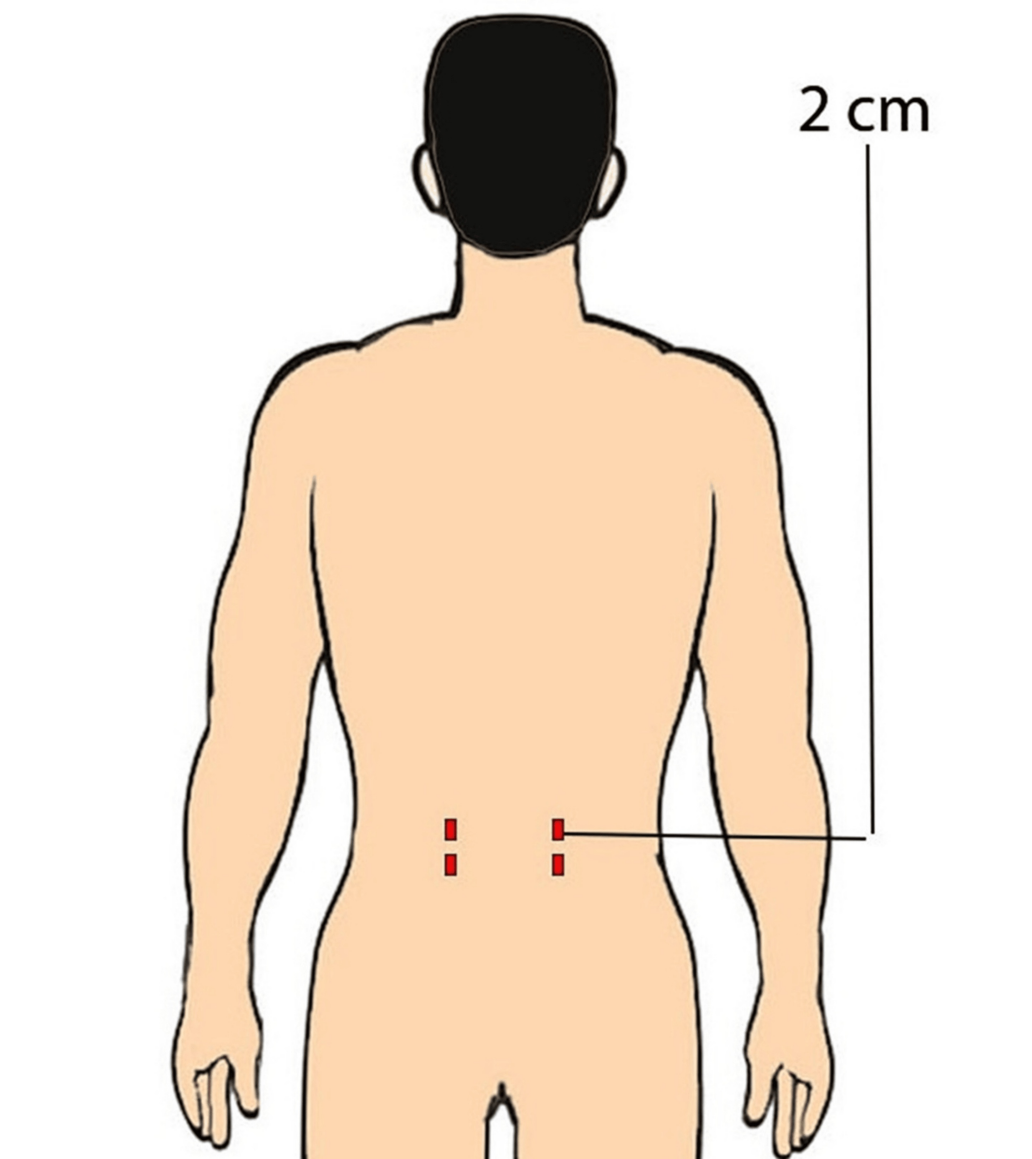
An animated picture showing multiple stab incision scars on the lower lumbar level

The sample size was identified using Statulator (Statulator, Evanston, IL) [19]. No studies in the literature assess scar preference in subjects before spine surgery; therefore, the proportion of patients preferring either scar type is still being determined. Assuming 50% of patients will prefer a midline scar and the other 50% a paramedian scar, a sample size of 385 was calculated to be necessary for estimating the expected proportion with 5% absolute precision and 95% confidence. The questionnaire provided demographic data, such as age, sex, job type, and social, geographical, and educational level. Participants were also given two colored illustrations (Figures 1-2), each with a different scar from lumbar spinal surgery (SOST and MIST). Respondents were asked which scar they thought was less visible, more aesthetically pleasing, and what they would want if they were to undergo lumbar spine surgery. To achieve an unbiased and precise data collection, the questionnaire was given independently by four trained personnel who were supervised till the completion. They were available and ready to address participants’ queries while filling out the form, which improved data clarity and quality. Respondents moved through the questions in a one-way vertical scroll, and no “back” or “return to the previous question.” No corrections were allowed.

Statistical analysis

The statistical analysis for this study was conducted using IBM SPSS Statistics version 2020 (IBM Corp., Armonk, NY). Mean, standard deviation, and minimum/maximum were calculated to summarize and describe the central tendencies and variability within the dataset for descriptive statistics. The chi-square test was utilized to investigate the associations and relationships between categorical variables, such as scar preferences and demographic characteristics. The Pearson test, a measure of the strength and direction of associations between two continuous variables, was employed wherever applicable. The Z-test was used to compare sample statistics with known population parameters, such as the mean or the proportion. p-values were computed to determine the significance of the observed associations and differences. The significance level was set at p < 0.05.

## Results

**Figure 3:**
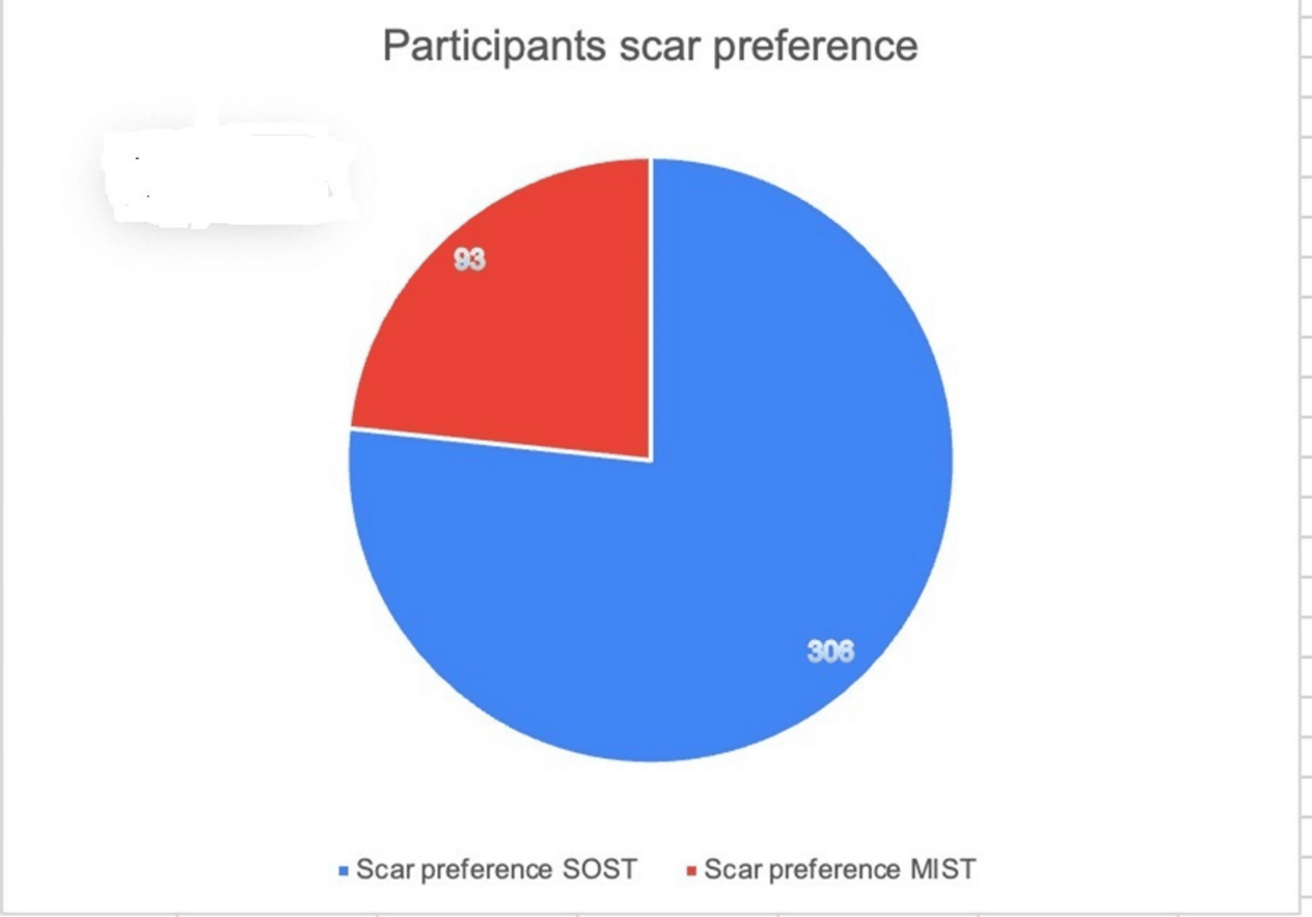
Pie chart showing the comparison between the standard open surgical technique (SOST) and minimal invasive surgical technique (MIST) scar preference

A total of 399 participants were enrolled in the study. The mean age of the participants was 33.58 ± 12.82 (18-77) years, with 229 females and 170 males. A Z-test for proportion yielded a highly significant result, as 306 selected the SOST scar, and 93 selected the MIST scar (Z = 17.725, p < 0.001) (Figure 3). Among all, 248 had no reported scars on their bodies, while 151 had pre-existing scars. 238 respondents expressed aesthetic concerns about the appearance of a potential scar, and 107 reported attempts to conceal blemishes on their bodies. Concerning the anticipation of acquiring scars after any surgery, 212 expressed concern, while 187 did not share this concern.

**Figure 4:**
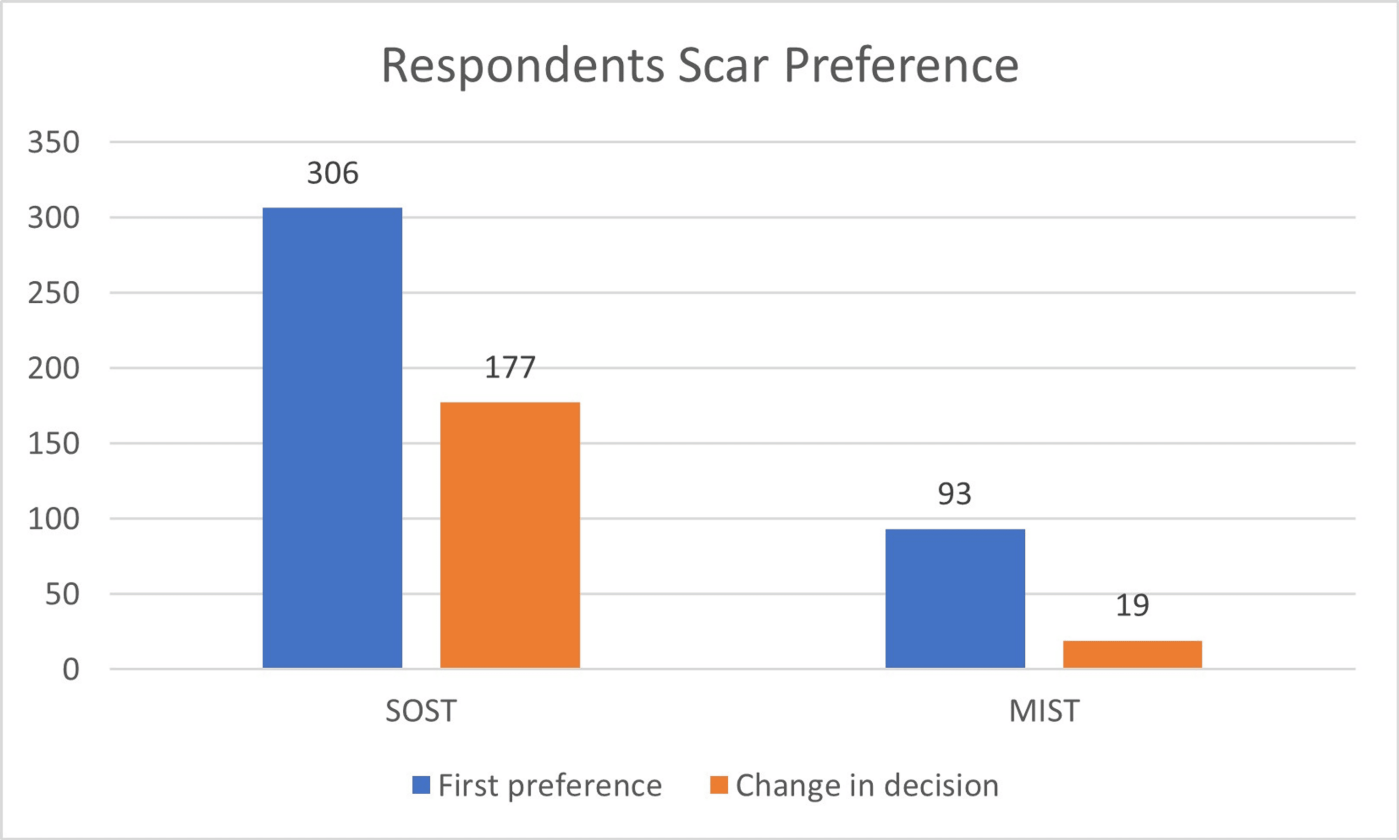
Bar diagram showing shift and preferences of respondents for back surgery scar standard open surgical technique (SOST) and minimal invasive surgical technique (MIST)

Regarding pre-existing back conditions, 128 were reported, while 271 had none. A statistically significant difference was found in participants who changed their decision (n = 139) after being told that SOST is an older method of surgery (p < 0.001). Regarding future choices, 354 indicated they would prefer the advanced modern surgical option MIST if they needed a lower back operation (Figure 4). Among those worried (n = 212) about scars in general (n = 176, 83.01%), favored SOST scars. For those not concerned about scars (n = 187), significant respondents (n = 130, 69.51%) still preferred SOST scars. Table 1 lists the demographic data of participants and their responses to the study questionnaire. Table 2 illustrates the substantial correlations between participants’ preferences for surgical scars, specifically the SOST and MIST techniques, and various demographic characteristics. Table 3 categorizes the noteworthy relationships between the impact of scars on individuals’ personal and professional lives (categorized as yes or no) and various demographic factors. A Z-test for proportion yielded a highly significant result, stating that the majority (n = 153, 38.3%) of the respondents were least concerned about the scar at the back when compared to other body scars (n = 246, 61.7%). Facial scar was the greatest concern among scars (n = 354, 89.22%).

**Table 1:** Demographic data and summary of participant responses MIST - minimal invasive surgical technique, SOST - standard open surgical technique

Patient Variable	Result
Age (years)	33.58 + 12.82 (13-77)
Gender	
Male	N = 170 (42.6%)
Female	N = 229 (57.39%)
Participants who tried to hide their scar	
Yes	N = 107 (26.81%)
No	N = 292 (73.18%)
Concern of scar on back compared to other body parts (scored between 1 and 5)	
1	N = 8 (2%)
2	N = 50 (12.53%)
3	N = 71 (17.79%)
4	N = 117 (29.32%)
5	N = 153 (38.3%)
Concern of scar after surgery	
Yes	N = 212 (53.13%)
No	N = 187 (46.86%)
Lower back problem	
Yes	N = 128 (32.08%)
No	N = 271 (67.91%)
Scar preference	
SOST	N = 306 (76.69%)
MIST	N = 93 (23.3%)
Advanced surgery preference	
Yes	N = 354 (88.72%)
No	N = 45 (11.27%)
Spine surgery scar’s perceived impact on professional and personal life	
Yes	N = 174 (43.6%)
No	N = 225 (56.39%)
Socio economic status	
Upper class elite	N = 28 (7.01%)
Upper class	N = 83 (20.8%)
Middle class	N = 160 (40.1%)
Lower middle class	N = 33 (8.27%)
Working class	N = 82 (20.55%)
Poor class	N = 13 (3.25%)
City	
Tier 1 city	N = 223 (55.88%)
Tier 2 city	N = 176 (44.11%)
Education	
High school	N = 200 (50.12%)
Graduate	N = 140 (35.08%)
Postgraduate	N = 50 (12.53%)
PhD	N = 9 (2.25%)

**Table 2:** Significant associations between scar preference (SOST and MIST) and demographic factors DF - degree of freedom, MIST - minimal invasive surgical technique, SOST - standard open surgical technique

Category	SOST (n = 306)	MIST (n = 93)	Total	Chi-square	DF	p-value
Education						
High school	143	57	200	13.964	3	0.003
Graduate	119	21	140	-	-	-
PhD/medical doctor	4	5	9	-	-	-
Postgraduate	40	10	50	-	-	-
Work						
No	63	22	85	8.766	2	0.012
Student	51	27	78	-	-	-
Yes	192	44	236	-	-	-
Living status						
Lower middle class	18	15	33	30.007	5	<0.001
Middle class	139	21	160	-	-	-
Poor class	12	1	13	-	-	-
Upper class	55	28	83	-	-	-
Upper class (elite)	25	3	28	-	-	-
Working class	57	25	82	-	-	-

**Table 3:** Significant associations between scar impact on professional or personal life and demographic factors (yes or no) DF - degrees of freedom

Category	No (n = 271)	Yes (n = 128)	Total	Chi-square	DF	p-value
Work						
No	55	30	85	10.295	2	0.006
Student	32	46	78	-	-	-
Yes	138	98	236	-	-	-
Living status						
Lower middle class	16	17	33	12.732	5	0.026
Middle class	105	55	160	-	-	-
Poor class	7	6	13	-	-	-
Upper class	37	46	83	-	-	-
Upper class (elite)	18	10	28	-	-	-
Working class	42	40	82	-	-	-
Age						
Adolescent	5	11	16	12.234	2	0.002
Adults	186	153	339	-	-	-
Old age	34	10	44	-	-	-

We noted a significant association between scar preference and the respondent’s socioeconomic status, employment status, and level of education, as depicted in Table 2.

## Discussion

The field of spine surgery has experienced substantial improvements, limited not just to imaging, anesthesia, surgical techniques, instrumentation, technology, documentation, standardization, and education that have resulted in better outcomes [20-22]. Nevertheless, surgeons must consider scar outcomes and disclosure to the patient to consider both clinical outcomes and legal issues [23]. Multiple variables may contribute to suboptimal surgical scar aesthetics in spine surgery. Typically, spine surgery is more about the patient's condition/injury with less emphasis on cosmetic scarring concerns. Forecasting a scar's outcome is tricky, and unique patient variables preclude any assurance of an exact cosmetic plan. Pain control and improved function are more important for many patients than looks. Scar outcome is not the main topic of conversation in spine surgery, yet it can be a critical concern from both patients' and surgeons' perspectives [16,24].

Participants in the study had similar spine surgical scar preferences and showed concern about their scar (n = 212, 53.13% ) irrespective of whether they had any lower back problem (n = 128, 32.08%) or not (n = 271, 67.91%). Interestingly, most participants (n = 306) favored the SOST scars, potentially perceiving them as less noticeable (Figure 3). This preference may be due to the para-median scar being a more visible and non-median location associated with multiple incisions of MIST techniques. Ricardi et al. compared aesthetic results following MIST (n = 74) and SOS (n = 44) for the lumbar spine, favoring the preference for a single midline incision statistically; this study assessed the grading of scars following surgery in both groups. Therefore, the option in question was not offered to the group of patients above before their surgery, and they needed to select it actively [16]. Hamouda et al. conducted a prospective study comparing the outcomes of a traditional vertical incision with a transverse incision in micro lumbar decompression. The results of their study indicated a statistical preference for the transverse incision approach [25]. A contrasting outcome is reported in a study with 106 participants to investigate patient opinions regarding the MIST skin incision illustration. The study compared traditional lumbar paramedian MIST skin stab incisions and other novel incisions. 76% (n = 65) expressed negative opinions about traditional midline incisions, with the majority favoring traditional stab incisions (n = 41,39.8 %), followed by novel larger intersecting vertical incisions (n = 37, 35.9 %). The less popular choices were the unknown horizontal ( n =20, 19.4 %) and mini-oblique (n = 5, 4.8 % ) incisions [17]. Surgeons can improve patient satisfaction by minimizing scar visibility and enhancing overall cosmesis via proper surgical technique, including incision choice and closure [26,27]. Several methods have been proposed, including perfect closure and others, including biodynamic excisional skin tension or Langer lines as incision guides to minimize postoperative scarring [28-31]. Although the novel horizontal and oblique incisions, aligned more closely with Langer's lines, offered advantages such as enhanced surgical access, reduced operative duration, and minimized radiation exposure, Quiring et al. found it less cosmetically preferable. This observation is particularly pronounced among female patients [17]. In 1968, a significant milestone in surgical technique emerged with the introduction of bilateral vertical incisions, positioned 30 mm from the midline, for Wiltse's paraspinal muscle-sparing approach. While this approach garnered considerable support, aesthetic concerns and potential complexities in cases of iterative surgery prompted a shift. In 1988, even Wiltse advocated transitioning to a single median incision. This historical transition demonstrates the nuanced evolution towards a midline approach, preceding the contemporary trans-tubular paramedian minimally invasive surgical method [32].

After learning that the SOST scar was an older surgical technique, a significant shift in preference occurred among the 306 participants who initially preferred it. Of those, 42.15% (n = 129) still favored the SOST scar, while 57.84% (n = 177) changed their preference to the MIST scar. Among the 93 who initially chose the MIST scar, a minor shift occurred when they learned that MIST was an older technique. In this group, 20.43% (n = 19) changed their preferences for the SOST scar, while 79.56% (n = 74) preferred the MIST scar. A significant shift (p < 0.0001) was noted in the SOST group as compared to the MIST group after being informed about the advancements in the surgical technique.

Regardless of age, study participants exhibited similar aesthetic preferences for SOST scars. Thus, the participants who expressed concern about surgical scars (n = 212, 53.13% ) or not (n = 187, 46.86%) exhibited SOST scar preferences.

A substantial majority of participants (n = 354, 89%) expressed an interest in additional benefits like faster recovery, better maintenance of natural bone and muscles, reduced pain, and advanced technology in surgical outcomes, in addition to better scarring. Only 45 (11%) respondents indicated they were not seeking these additional benefits and were primarily focused on scar aesthetics. These groups of patients need to be identified before surgery, as they may indirectly become poor outcome candidates or even medicolegal inciters.

Individuals without current employment preferred the SOST scar (p = 0.006), indicating a unique aesthetic preference. Lower-middle-class participants (p = 0.026) also significantly favored the SOST scar. Moreover, adolescents (p = 0.002) especially preferred the SOST scar, emphasizing age's influence on choices. The participant sample featured approximately equal gender homogeneity, which improves generalizability for both males and females. The information about the occupations showed a rich mix of educational backgrounds, which enriches the study and makes its results generalizable to different education levels. Contrastingly, our study also noted that most respondents (n = 153, 38.3%) were least concerned about the scar at the back and, expectedly, most about facial scars. 

Many scoliosis patients did not swim or go to the beach because they did not want anyone to see the scar. Getting changed in and out of sportswear seemed equally mortifying, underscoring the highly negative mental and social impact that spine surgical scars can have [12-14]. Some studies are available to discuss the significance of improved cosmesis in transverse cervical anterior exposure compared to traditional vertical incisions [33,34]. In a randomized controlled trial comprising 530 participants with the lumbar spine, it was observed that individuals who received percutaneous transforaminal endoscopy exhibited more favorable outcomes in terms of smaller scar size and scar-related measures (including body image scale, cosmesis score, and the numerate rating scale) as compared to those who underwent open microdiscectomy [18]. These findings highlight an intriguing aspect of patient preference regarding surgical outcomes, emphasizing the importance of considering clinical and functional results and aesthetic considerations in surgical decision-making [35]. Techniques that enhance aesthetic outcomes without compromising clinical effectiveness could be worth exploring further in lumbar spine surgery.

Limitations

No established standards existed to serve as a reference for developing our questionnaire. We used illustrated photos of scars from lumbar spine surgeries rather than actual scars, which may limit the study's external validity. Still, a representative image of MIST and SOST could be given, though it cannot be generalized for all lumbar back posterior surgeries. The study should have followed up with actual patients operated on to assess the long-term satisfaction with their scars. It would have been more valid, but patients who underwent spine surgery were excluded. This was ethically impossible as it would have created conflict in our patient's mind, as the same issue was not discussed extensively pre-operatively. Respondents in the study may have been more conscious of their appearance if they did not have any spinal disorder, which may have affected their preferences for scars. This self-selection bias may limit the generalizability of the study to the larger population of patients undergoing lumbar spine surgery. Patients may have different experiences and expectations regarding scarring depending on their individuality, social strata, region of the country, or even the Western world, which may affect their preferences, which needs multi-centric data and an extensive population study. The number of participants in the current study is small. However, we used a valid statistical tool, Statulator, to calculate the minimum requirement. The sample size (399) exceeded the suggested 385 minimum recruitment.

Moreover, our representative sample has been from only two cities (residents and non-residents), and the people approached were from hospitals, accompanied by patients, and other public places till a minimum number target was reached. So, only some of the population groups are included. There can be a bias, but the primary author and surgeons were not involved in data acquisition to avoid such discrimination. Scars in different areas of the body, whether larger or smaller, may be perceived differently by patients, which may have affected the study results. Participants may have been influenced by response bias, where they may have felt pressure to provide socially desirable responses or may have responded in a way they thought would please the researcher, even though no attempt was made to bias the questionnaire. The questions contained blanket adjective terms like advanced surgery, which do not objectify the type of surgery or each aspect of its advantages. This is the first study that has done such a notional study. Many unique factors may have yet to be fully accounted for in the study, limiting the validity of the results.

## Conclusions

The study highlights the significance of considering patient-oriented aesthetic outcomes in lumbar spine surgery. The concern of back scars is less compared to other body scars. However, given a choice, the midline scar is perceived as having lower visibility and better aesthetics. A modern advanced technique is preferred, and scar takes a backseat if the advantages are discussed. These findings underscore the need for spine surgeons to prioritize discussions on scar outcomes with patients, considering the increasing penetration of minimally invasive techniques. This study advocates for a patient-centered approach that balances clinical efficacy with patient satisfaction, ultimately enhancing spine surgery's overall quality of care.
